# Osteoimmunology in Periodontitis: Local Proteins and Compounds to Alleviate Periodontitis

**DOI:** 10.3390/ijms23105540

**Published:** 2022-05-16

**Authors:** Kridtapat Sirisereephap, Tomoki Maekawa, Hikaru Tamura, Takumi Hiyoshi, Hisanori Domon, Toshihito Isono, Yutaka Terao, Takeyasu Maeda, Koichi Tabeta

**Affiliations:** 1 Division of Periodontology, Graduate School of Medical and Dental Sciences, Niigata University, Niigata 951-8514, Japan; kridtapat.s@dent.niigata-u.ac.jp (K.S.); h-tamura@dent.niigata-u.ac.jp (H.T.); koichi@dent.niigata-u.ac.jp (K.T.); 2 Center for Advanced Oral Science, Graduate School of Medical and Dental Sciences, Niigata University, Niigata 951-8514, Japan; hiyoshi@dent.niigata-u.ac.jp (T.H.); maedat@dent.niigata-u.ac.jp (T.M.); 3 Faculty of Dentistry, Chulalongkorn University, Bangkok 10330, Thailand; 4 Division of Microbiology and Infectious Disease, Graduate School of Medical and Dental Sciences, Niigata University, Niigata 951-8514, Japan; hisa-domon@dent.niigata-u.ac.jp (H.D.); tisono@dent.niigata-u.ac.jp (T.I.); terao@dent.niigata-u.ac.jp (Y.T.)

**Keywords:** periodontitis, osteoimmunology, bone regeneration, osteocytes, immune cells, RANKL

## Abstract

Periodontitis is one of the most common oral diseases resulting in gingival inflammation and tooth loss. Growing evidence indicates that it results from dysbiosis of the oral microbiome, which interferes with the host immune system, leading to bone destruction. Immune cells activate periodontal ligament cells to express the receptor activator of nuclear factor kappa-B (NF-κB) ligand (RANKL) and promote osteoclast activity. Osteocytes have active roles in periodontitis progression in the bone matrix. Local proteins are involved in bone regeneration through functional immunological plasticity. Here, we discuss the current knowledge of cellular and molecular mechanisms in periodontitis, the roles of local proteins, and promising synthetic compounds generating a periodontal regeneration effect. It is anticipated that this may lead to a better perception of periodontitis pathophysiology.

## 1. Introduction

The human oral cavity contains many microbial species constituting commensal bacteria, with interactions between the host and oral microbiota defining oral cavity health. The oral microbiota is a distinct and diversified ecosystem of microbial organisms that each microorganism interacts with, metabolically and physically. These complex interactions culminate in the creation of biofilm communities in which physiochemical gradients establish diverse habitats for microorganisms requiring varying metabolic activities [1]. The microbiota structure in healthy supragingival plaque resembles the “hedgehog-like” network, resulting from the radially spatio-chemical gradient [2]. In the structure depicting the plaque microbiota described by Welch et al. [2], early colonizers, such as *Actinomyces* spp. and *Streptococcus* spp., adhere to the tooth surface via interaction of non-specific and specific binding adhesins on their cell surfaces and salivary proteins within the pellicle [3]. Consequently, *Corynebacterium* spp. attaches to the early colonizers and radially grows exteriorly to form a long, annular structure. *Haemophilus*, *Aggregatibacter*, and *Neisseriaceae* attach to the tip of the annulus owing to their metabolic activity requiring the abundance of both oxygen and nutrients. Metabolic products from oxidative species at the periphery create anoxic circumstances in the core of the biofilm, where anoxic capnophilic species, such as *Capnocytophaga* and *Fusobacterium*, prefer to grow [2]. Unlike many infectious diseases, periodontitis results from oral microbiota dysbiosis [4,5,6]. The etiology of periodontal diseases is due to periodontal-pathogenic bacteria. When the sophisticated interactions of the oral microbiota are disturbed by the keystone bacterium, *Porphyromonas gingivalis*, the polymicrobial dysbiosis transpires [7,8]. This disturbance in plaque microbiota is a significant etiology of gingival inflammation causing the onset of periodontitis [8,9,10]. Oral microbiota dysbiosis interferes with the host immune system, resulting in inflammatory conditions, in turn leading to cellular interactions of immune and bone cells and subsequent bone destruction [11,12]. The cellular interactions and molecules related to the communication between immune and bone cells have gained popularity in recent years, providing more insights into periodontitis.

Osteoimmunology focuses on the cellular and molecular mechanisms behind inflammatory bone resorption, which destroys alveolar bone [13]. Recent studies in osteoimmunology focused on understanding the pathogenesis and development of therapies for inflammatory bone diseases, such as rheumatoid arthritis and periodontitis [14]. This review discusses the cellular events related to periodontitis pathophysiology and the local proteins and synthetic agents, which are expected to be the future therapeutic targets to alleviate this condition.

## 2. Periodontitis: Immune Response and Microbiota

### 2.1. The Novel Classification of Periodontal Diseases (the AAP 2017 Classification)

The American Academy of Periodontology (AAP) has given several classifications of periodontal disease over the past 40 years. In the AAP workshop in 1989, the committee categorized periodontitis as follows: prepubertal, adult, and juvenile periodontitis (distribution as localized and generalized) and rapid progression referred to the clinical manifestations, age of onset, and rate of progression [15]. The periodontitis classification that was used for almost two centuries, and has been referred to later in the text, was significantly revised in 1999. In the 1999 AAP classification, periodontitis was classed as a necrotizing, chronic, aggressive (distribution as localized and generalized), and systemic disease presentation [16]. Recently, epidemiological studies and basic scientific research analyzing environmental and systemic risk factors have led to a better understanding since the latest modification in the diagnosis and classification of periodontitis.

In view of recent data, a joint workshop of the AAP and the European Federation of Periodontology (EFP) was conducted in 2017 to revise and create a novel scheme for the classification of periodontitis [17]. At this workshop, it was agreed to classify three forms of periodontitis: (1) necrotizing periodontitis; (2) periodontitis as a manifestation of systemic disease; and (3) periodontitis, the form of periodontitis, formerly known as “chronic” or “aggressive” periodontitis [18]. Moreover, in the workshop, a procedure for categorizing the severity of periodontitis using the staging and grading system was decided, which could be modified over time with multidimensional disease progression of periodontitis [19]. The staging system is subject to the severity of the disease measured using clinical parameters, such as clinical attachment level (CAL), recession, and intricacy of case management. Whereas the grading system represents supplementary data on the nature and characteristics of current pathology, including the patient history is taken to assess the progression rate, estimation of any risks for future progression, predicted complications of treatment, and evaluation of individual factors that could be compromised by periodontal treatment. 

The staging comprises four classes—stage 1, the early phase of attachment loss, through stage 4, characterized by significant damage to the periodontium, followed by extensive tooth loss and loss of masticatory function. There are three grading levels—A, B, and C, representing low, moderate, and high-risk progression, respectively. Apart from disease progression, grading is determined by other factors related to disease progression, including individual health conditions and risk factors worsening the disease and treatment outcomes, such as smoking or uncontrolled diabetes. Therefore, grading authorizes clinicians to integrate patient characteristics into the diagnosis required for comprehensive patient care [17,19]. Furthermore, a new category of periodontal health in the intact periodontium was introduced, which is supposed to be a goal for treatment outcomes and sustainable conditions in the maintenance phase. With the skyrocketing popularity in implant dentistry, this new classification also launched peri-implant health, mucositis, peri-implantitis, and soft- and hard-tissue deficiencies around implants [20]. Additional notable points of the new classification were as follows [17]: (1) replacement of the previous terms “biological width” and “periodontal biotype” with newly introduced terms “supracrestal attachment” and “periodontal phenotype”, respectively; (2) addition of new terms “gingival pigmentation,” “traumatic occlusal force,” and “periodontitis as a manifestation of systemic disease;” (3) emphasis on smoking, together with diabetes, as significant risk factors for grading the disease; (4) management procedure of gingival recession, established on the interproximal attachment loss; and (5) when unrelated to the dental plaque, systemic diseases affecting the periodontium are now categorized as “Systemic Diseases or Conditions Affecting the Periodontal Supporting Tissues.”

This introductory summary briefly highlights the modifications that have been made to the 1999 classification of periodontal disease. It does not illustrate the overall comprehensive information in the literature, additional consensus reports, and case definition writings, which have guided the launch of this latest classification. Nonetheless, the reader could use the information presented in this section to understand the notable changes in the 1999 classification, as an adjunct to the complete reports on which there is a consensus, to manage patients properly or to conduct further scientific investigations.

### 2.2. Roles of Th17 and exFoxp3Th17 Cells in Periodontitis

**Figure 1:**
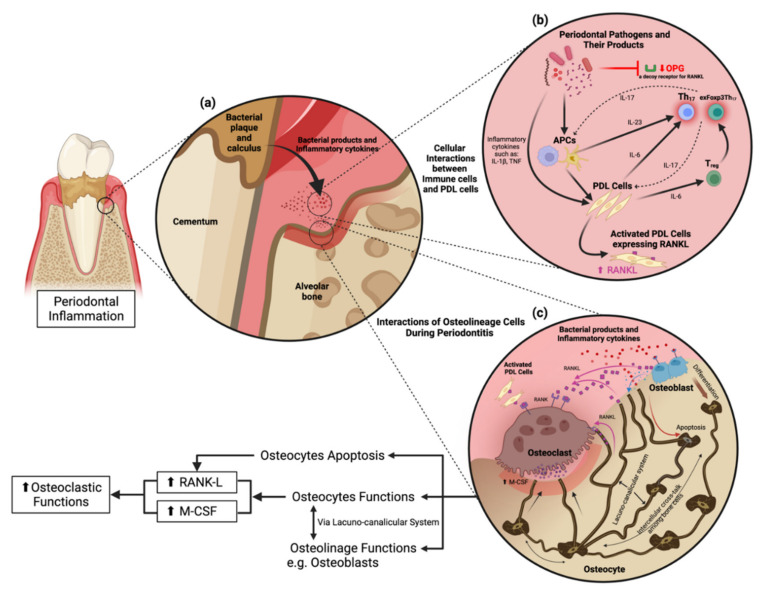
Cellular interactions and osteocyte function in periodontitis. (**a**) Pathogenic bacteria in the periodontal pocket release their virulence factors to stimulate proinflammatory cytokine production from periodontal stromal cells and immune cells in the periodontium. (**b**) Cellular interactions in response to periodontitis. Periodontal pathogens and their products activate antigen-presenting cells (APCs) in the periodontium to stimulate periodontal ligament cells (PDL cells) following the activation of Th17 and exFoxp3Th17 cells. (**c**) Osteocyte functions and their interactions with other cells through the lacunocanalicular system (LCS).

Th17 cells are the CD4^+^ T cell subset that stimulates osteoclast differentiation. In contrast, Foxp3-expressing regulatory T (Treg) cells suppress inflammation and inhibit osteoclastogenesis [21]. Immune cells in arthritis-autoimmune disease have a subset of Treg cells that transform into interleukin-17 (IL-17)-expressing Foxp3 T cells (or exFoxp3Th17 cells) and exhibit Th17 cell-like functions following inflammatory stimuli. In addition, exFoxp3Th17 cells activate the osteoclastogenic activity more strongly than the conventional Th17 cells. Consequently, exFoxp3Th17 cells are critical for osteoclastogenesis [22]. These two T cell subsets are related to periodontitis and occur in periodontitis lesions [23]. Mice lacking Th17, exFoxp3Th17 cells, and IL-17 show a subtle inflammatory condition during periodontitis with less alveolar bone loss [23,24]. Moreover, human T cells expressing IL-17 have more periodontitis sites [23]. Foxp3^+^IL-17^+^ T cells are significantly observed in severe periodontitis lesions and are suggested as the cells in transition from Treg cells to exFoxp3Th17 cells [25]. In summary, both Th17 cells and exFoxp3Th17 cells are strongly implicated in periodontitis (Figure 1).

### 2.3. Cellular Interaction to Orchestrate Inflammation

A healthy periodontium maintains a sophisticated dynamic equilibrium of inflammatory cytokines to control inflammation. Periodontal tissue destruction occurs when this equilibrium is changed in favor of proinflammatory cytokines. Neutrophils are major players in periodontitis and other chronic inflammatory disorders, including rheumatoid arthritis, atherosclerosis, diabetes, and cancer [26,27]. Furthermore, neutrophils are responsible for the severe destruction of alveolar bone, and their numbers are strongly associated with periodontitis severity [28,29,30]. Accordingly, periodontitis pathogenesis is inevitably related to the inflammatory cytokine cascade and the crosstalk between immune and periodontal cells. Periodontitis-related inflammatory cytokines, such as tumor necrosis factor (TNF) and IL-1β, produced from host cells exposed to the dental biofilm, produce a synergistic effect by increasing IL-6 synthesis resulting in increased Th17 cell activation [31]. The activation of both Th17 and exFoxp3Th17 depends on IL-6 production from stromal cells in the periodontal tissue, such as periodontal ligament cells (PDL cells) [24]. Th17 cell expansion-related periodontal inflammation also depends on the activation of the local microbiome by IL-6 and IL-23 [23].

### 2.4. Roles of Interleukin 6 and Its Receptors in the Development of Periodontitis

An inflammatory reaction causes the destruction of periodontitis-affected tissue. Among the primary proinflammatory cytokines associated with periodontitis, interleukin (IL)-6 promotes the cascade of destructive tissue processes [32]. In the group of the IL-6 family identified regarding the mutual usage of receptor chain gp130 (CD130) [33], IL-6 is involved in the pathogenesis of periodontitis [34]. IL-6 binds directly to the transmembrane receptor (mIL-6R) found in monocytes, lymphocytes, etc. [35], or to its soluble form (sIL-6R) after mIL-6R is cleaved by specific proteases [36]. The binding of IL-6 and its receptor, IL-6R, enables the dimerization of GP-130, triggering the Janus kinase (JAK)/signal transducer and activator of transcription (STAT) and MAPK signaling pathways [37].

IL-6 is a multifunctional cytokine having a substantial impact on periodontal tissue damage. Its relevance in the early phase of periodontitis development has been studied [34,35]. The IL-6 174G/C polymorphism increases the risk of developing periodontitis [38]. Moreover, according to a meta-analysis, chronic periodontitis patients have increased IL-6 levels in their gingival crevicular fluid, whereas there is no change in the levels following the hygienic phase of the periodontal treatment [39]. In an in vivo study on non-human primates, IL-6 expression was increased during the early phase of periodontitis development, whereas the expression remained low during the progression of periodontitis and during the resolution period after the elimination of inflammation [40]. These findings demonstrate the essential roles of IL-6 in periodontal tissue damage at the beginning and in the acute phases of periodontitis development.

The levels of both IL-6 and its receptors are elevated in the inflamed periodontium, for example, gingiva, gingival crevicular fluid, and even in the blood plasma [41]. In the resolution phase, the level of IL-6 in the blood serum was reduced following periodontal therapy [42]. In experiments on mice, inhibition of IL-6-influenced inflammation by blocking its receptor could reduce alveolar bone loss, supported by alterations in Th17 responses to periodontitis [43]. If the exogenous soluble receptor of IL-6 (sIL-6R) is found abundantly in the gingiva, IL-6 might bind to gingival fibroblasts (GF) [44,45]. The binding of IL-6 and its soluble type of receptor (IL-6/sIL-6R) resulted in a dose-dependent increase in the expression of the gene encoding matrix metalloproteinase (MMP-1), an enzyme that degrades the bone matrix and proteins [45]. In cultures of gingival fibroblasts, IL-6/sIL-6R also increased the collagenolytic activity. However, an IL-6/sIL-6R mixture did not affect cell proliferation or the expression of tissue inhibitor of matrix metalloproteinase (TIMP) gene, either alone or in combination [44]. IL-6 affects osteoclast functions both indirectly and directly. IL-6 indirectly improves osteoclast formation by elevating the production of receptor activators of nuclear factor kappa-B ligand (RANKL) from osteoblasts or stromal cells in the PDL space. IL-6/sIL-6R also directly regulates RANKL-mediated osteoclastogenesis and osteoclast functions via the NF-κB, ERK, and JNK signaling pathways [37]. Although the binding of IL-6 and its soluble form receptor affected the osteoclasts, IL-6-influenced osteoclast formation was incomplete when the soluble form of IL-6 receptor (sIL-6R) was lacking because osteoclastogenesis cannot occur in the absence of the soluble form receptor [46], revealing the insufficiency of the membrane form of IL-6R expressed on the osteoclast surface.

All these results emphasize the importance of IL-6 and its transmembrane (mIL-6R) and soluble (sIL-6R) receptors in the acute phase of periodontitis and in tissue destruction by elevating the activity of enzymes. Blocking the IL-6 receptor results in attenuation of periodontitis in vivo. Besides, only sIL-6R has a substantial effect on osteoclastogenesis and mIL-6R does not promote osteoclastogenesis in vitro. These results raise the significance of IL6 and its receptors as possible targets for eliminating inflammation, mitigating tissue destruction, and preventing osteoclastogenesis, which can prevent further tissue destruction that follows the crosstalks of immune cells and periodontium as partial consequences of the IL-6/IL-6R binding.

### 2.5. “Polymicrobial Synergy and Dysbiosis” Concept

The communication among the oral microbiota, the host immune system, and the osteolineage cells related to the progression of periodontal diseases is sophisticated. Such complexity occurred regarding the diversity of bacteria in the microbiota in the subgingival area and the microbiome variation of the host [7,47]. Besides, the complexity of the immunological responses of the host functioning beneath the periodontium is sometimes ambiguous whether these responses are considered therapeutic or destructive resulting from the uncontrolled immune responses [48]. Despite these complications, previously established principles have already defined the roles of the microbiota in the development of periodontitis. 

Bacteria are required at the onset of the destructive disease. Studies on germ-free animals and therapeutic approaches targeted at subgingival biofilm have established that a commensal microbiome is obligated for violent alveolar bone loss in periodontitis [10]. Furthermore, transformations in the whole microorganism population, or dysbiosis, and their density in subgingival biofilms correlate with the destruction level of periodontitis. Even though the microorganisms in the biofilm have been analyzed extensively and contain approximately 150 to 800 species identified within the human dental plaque [49], the dispute over whether species are particularly virulent and capable of initiating periodontitis has persisted for years. Although the currently well-known pathogens are gram-negative anaerobic bacteria and spirochetes, no specific pathogens may independently be accountable for the disease. Instead, the dysbiosis (the dysequilibrium in the microbial biofilm) turns into the “pathogenic unit” [50] or “dysbiotic microbiota” [51], leading to gingival inflammation and periodontitis. This dysbiosis results from the colonization of the periodontal microbiota by the keystone pathogens (e.g., *Porphyromonas gingivalis)* and accessory pathogens, oral commensal bacteria with the ability to enhance keystone pathogen metabolic activity and colonization (e.g., *Streptococcus gordonii*) [7]. The colonization of keystone pathogens aided by accessory microbiota results in component and numerical modifications that allow for the development of dysbiosis in a susceptible host [28]. When the homeostasis between a microbe and host is disrupted, bacteria attracted to inflammation called “inflammophilic pathobionts” overwhelm and further promote inflammatory destruction [51]. The disturbance of homeostasis in the oral microbiome is a prerequisite for the outbreak of inflammatory destruction and persistent tissue damage to occur [7]. However, dysbiotic microbiota generated by inflammation is not limited to the overgrowth of pathogenic species. Additionally, growth circumstances produce an environment that alters the virulence factors of the biofilm community and increases their pathogenicity. Transcriptomic studies of the microflora associated with progressive periodontitis indicated that pathogenic and normal commensals mutually upregulate the expression of virulence factors [52].

At relatively low colonization numbers, *P. gingivalis* affects the constituents and density of the oral commensal microbiome, resulting in inflammatory alveolar bone loss. In oral epithelial cells, *P. gingivalis* promotes the Notch 1 signaling pathway, leading to the synthesis of PLA2-IIA (PLA2G2A), an antimicrobial protein that later induces dysbiosis [53]. *P*. *gingivalis* also manipulates the host-protective pathway, which activates small inflammatory responses leading to chronic inflammation. *P*. *gingivalis* also inhibits the protective TLR2-MyD88 pathway in neutrophils through MyD88 proteasomal degradation, whereas it activates the alternative TLR2-Mal-PI3K pathway. The TLR2-Mal-PI3K pathway suppresses phagocytosis, protects other susceptible microorganisms, and induces dysbiotic-mediated inflammation in vivo [6]. These results reveal the importance of commensal bacteria in the pathogenesis of periodontitis, and particular species, such as *P. gingivalis*, could affect host-microbial ecology and culminate in alveolar bone resorption. 

This disease-related dysbiosis of the microbiome may be provoked by modifications in the surrounding environmental factors, such as the inflammation of the host, which has been ascribed as one of the possible causes accounting for dysbiosis in oral microbiota [23,24,54]. Although bacteria are known as the etiologic cause of gingivitis, the immunological reaction from the host to those organisms determines the progression of the illness. The accumulation of data shows that unregulated inflammation and host immunological reactions could be primarily accountable for destroying the alveolar bone in periodontitis [7,24]. Inflammation and dysbiosis develop mutually, and their interactions become the driving force for developing periodontitis in susceptible individuals [7]. Instead of just pathogenic bacteria being the etiology, the immunological response of the host leading to inflammation initiates tissue injury in periodontitis. This disease relies on mutually reinforcing connections between the host and the microbes [28]. All previous novel concepts have been incorporated into a new concept to describe the pathogenesis of periodontitis known as the “polymicrobial synergy and dysbiosis,” [28] which explains that periodontitis develops in susceptible hosts as a result of the sum of the cooperative pathogenicity of interactions between integrated microbiota mediated by keystone pathogen and a dyshomeostasis inflammatory response from hosts [7,8,28].

### 2.6. Roles of Neutrophils in Periodontitis

Neutrophils, the essential cells in innate immunity, have long been regarded as the homogeneous group of short-existed cells that predominantly participate in extracellular pathogen defense through phagocytosis and intracellular cell death [55]. Neutrophils have various weapons that assist in their roles in the immune system, including the formation of reactive oxygen species (ROS) and enzymes in the cytoplasmic granules of their cells [26]. Current data have contributed to a better understanding of the nature of neutrophils and their active functions within the periodontium, not limited to just the acute inflammation as was the perception. 

The previous concept of neutrophils being a homogeneous population of short-existed cells has changed with the advancements in laboratory technology. Different cellular markers have been discovered on the neutrophil surface, which allows the neutrophil to function in distinct roles classified as functional phenotypes [56]. Suppressive-type (CD62L- expression) decreased after periodontitis treatment, indicating that specific subtypes of neutrophils might be accountable for the progression of periodontitis. Moreover, periodontitis treatment could also alter distinct neutrophil phenotypes in peripheral blood samples during the first-year duration after treatment [57]. However, the role of this subtype of neutrophils in their involvement in peridontitis remains unknown and requires further studies.

Although neutrophils are associated with the early phase of inflammation in the initial progression of periodontitis or acute inflammation [58], these immune cells are now progressively revealed as significant effector cells in chronic diseases, such as periodontitis [30], rheumatoid arthritis [59], atherosclerosis [27], intestinal inflammation [60], and even in cancer [61]. As a result of chronic inflammation, in the oral tissues of chronic periodontitis patients, neutrophils live longer than those in healthy patients [62]. In periodontitis, neutrophils can cause tissue destruction in the periodontium due to their subversion by periodontitis-related microbes causing dysbiosis in the oral microbiota and, consequently, inflammation [10,28,47]. *P*. *gingivalis* entails the progression of periodontitis by exploiting complement receptors on neutrophils and macrophage surfaces. These keystone bacteria exhibit the ligands to activate the signaling from toll-like receptor-2 [6] and contain their gingipain enzymes (HrgpA and RgpB), which function close to C5 convertase leading to increased engagements of the C5a ligand [63,64]. Accordingly, neutrophils and macrophages are subverted due to this keystone bacteria-driven inflammation [65]. Therefore, *P. gingivalis* manipulates the functions of neutrophils through two different methods that, when integrated, allow the survival of the whole dysbiotic microorganism community and the continuation of the inflammation in the periodontium.

The link between neutrophils and inflammatory tissue damage, such as one in periodontitis, may entail processes other than the normal immune cells in the inflammation process. A novel concept arises as neutrophils are likely to be required for essential immunoregulatory roles. Appropriate neutrophils are necessary for the homeostasis of periodontal and other mucosal tissue [28]. The absence of this mechanism leads to hyperactive neutrophils in the body, followed by aggressive periodontitis in susceptible individuals [28,66]. The nearby connective tissue can be extensively injured by dysregulated neutrophils releasing inflammatory and toxic chemical substances, for example, ROS, extracellular-degrading matrix metalloproteinases, including collagenase enzyme, which leads to the progression of alveolar bone loss [67,68,69]. The effectiveness of innovative treatment methods that target proteins in the discovered pathways affirms their significance and they are speculated to be among the therapeutic agents for periodontitis treatment. The promising proteins for therapeutic approaches possessing immunomodulation effect will be discussed in a subsequent section with regard to the concept of “tissue-specific immunity.”

## 3. Periodontitis: Osteoimmunology

### 3.1. RANKL and OPG

Bone is a dynamic tissue resulting from calcium and collagen homeostasis through balanced osteoclast and osteoblast function. Osteoclasts derived from monocytes/macrophages are multinucleated cells that resorb bone matrix. Osteoblasts control osteoclastogenesis via the production of macrophage colony-stimulating factor (M-CSF) and RANKL, an essential cytokine in the activation of osteoclasts [14,24,70]. Alveolar loss results from dominant osteoclast activity. This activity depends on the interaction of three proteins composing the RANK/RANKL/OPG axis [71,72]. Transmembrane receptor activator of nuclear factor-κB (RANK) receptor is expressed in progenitor and mature osteoclasts with RANK binding to its ligand, RANKL, determining osteoclast activity. RANKL is a cytokine in the TNF family [13] that activates the RANK receptor [73]. However, osteoprotegerin (OPG) is a soluble decoy receptor for RANKL, impeding the RANK/RANKL binding and inhibiting osteoclastogenesis [74]. The RANKL/OPG ratio increases at periodontitis-affected sites, emphasizing the importance of the equilibrium between the molecules in this axis, especially RANKL and OPG levels [75,76]. 

Membrane-bound RANKL is responsible for almost all primary functions; however, soluble RANKL has a minor role in physiological bone homeostasis in mice [77]. Depriving soluble RANKL in the mouse model did not influence the level of bone destruction, which emphasizes the role of membrane-bound RANKL in the pathology of osteoporosis and periodontitis [24,77,78]. Accordingly, cells expressing membrane-bound RANKL are either near the bone surface or in contact with it [14]. exFoxp3Th17 cells exhibit the highest mRNA and protein levels of RANKL amongst T cell subsets [24]. Moreover, Th17 and exFoxp3Th17 cells produce IL-17 to activate mesenchymal cells, such as osteoblasts and PDL cells, to express RANKL on their membrane and produce proinflammatory cytokines to stimulate osteoclastic differentiation following alveolar bone loss [24,79]. Therefore, cells expressing membrane-bound RANKL derived near the periodontitis-affected bone surface lead to periodontitis. In addition, Th17 cells and exFoxp3Th17 T cell subsets likely contribute to mesenchymal cell stimulation via IL-17 to express RANKL, followed by stimulation of osteoclast functions.

Reduced OPG levels result in an elevated RANKL/OPG ratio, with a human study demonstrating significantly decreased *OPG* mRNA expression and OPG immunostaining in periodontitis lesions compared with the healthy periodontium [80]. In addition, proteases derived from oral bacteria and osteoclasts cleave OPG and stimulate osteoclast function in vitro [81,82]. This emphasizes the important roles of each protein in controlling osteoclast function in the axis, which is the critical activity affecting periodontitis.

### 3.2. Osteocytes Are Not Just Quiescent Resident Cells

Periodontitis is a multidimensional disease causing the tissue and teeth-supporting bone to disorganize. The pathophysiological process of periodontal disease has primarily focused on bone loss, particularly osteoclastogenesis and osteoblastogenesis. Recent studies show that osteocytes are not quiescent and contribute to physiological and pathological events in periodontitis [83].

Osteocytes are derived from an osteoblast cell lineage differentiated from mesenchymal stem cells (MSCs) in the bone marrow and account for most of the bone cells. They have an extended lifetime of up to 25 years compared with a half-life of 150 days for osteoblasts [84]. When osteoblasts cease to secrete extracellular matrix (ECM), they either undergo apoptosis or differentiate into osteocytes [85,86]. Communication between osteocytes and other cells can occur via dendrites reaching the surface of the bone (the lacunocanalicular system) [86]. This connection establishes crosstalk between osteoclasts, osteoblasts, bone lining cells, and bone marrow cells with the osteocytes [84,86,87]. Osteoclasts affect bone metabolism via osteocyte connectors. Osteoclast-derived leukemia inhibitory factor reduces sclerostin expression in osteocytes and subsequently promotes osteoblastic bone formation [88,89].

### 3.3. Osteocytes Induce Osteoclastogenesis via M-CSF (CSF-1) and RANKL

Osteoclasts are multinucleated giant cells originating from monocyte/macrophage cells and are accountable for bone resorption. Characteristics of periodontitis progression include alveolar bone loss resulting from osteoclast differentiation or osteoclastogenesis [90]. Osteoclastogenesis occurs via the production of colony-stimulating factor-1, also known as M-CSF, a factor required for osteoclast differentiation [91,92]. Continuous production of M-CSF in osteocytes enhances osteoclastogenesis [93]. Osteocyte-derived M-CSF protects against excessive Nox4-derived ROS generation and retains bone remodeling [94].

RANKL is a membrane-associated cytokine required for osteoclastogenesis synthesized by osteoblasts and osteocytes [91]. Osteoclastogenesis driven by RANKL is critical for inflammatory bone resorption, and its expression increases in periodontitis [95,96]. RANKL is synthesized by osteoblasts and interacts with the RANK receptor on the cell membrane of pre-osteoclasts to initiate conversion into active osteoclasts. OPG is the naturally present RANKL decoy receptor and is generated by various osteoblast lineages [97]. OPG competes with and reduces RANKL binding to RANK, inhibiting the activation of osteoclasts [98].

Osteocytes produce RANKL during bone remodeling in periodontitis (Figure 1). The concept of osteoblasts being the primary cellular source of RANKL has shifted toward osteocytes, which serve as the primary source of RANKL in bone remodeling instead of osteoblasts [99,100]. Gram-negative bacteria-derived lipopolysaccharide interacts with toll-like receptors on the osteocyte cell surface to stimulate the mitogen-activated protein kinase/extracellular signal-regulated kinase (ERK) 1/2 signaling pathway resulting in the activation of transcription factors that upregulate IL-6 expression [101]. IL-6 then activates Janus kinase through gp130, phosphorylating signaling molecules, and activator of transcription (STAT), leading to the translocation of STAT into the nucleus, enhancing RANKL expression in osteocytes [102,103]. Apart from IL-6, TNF-α is a typical inflammatory cytokine in periodontitis that directly stimulates osteocytes to produce RANKL and induce osteoclastogenesis or promote sclerostin expression in osteocytes leading to increased osteoclastogenesis [93]. Nevertheless, TNF-α does not induce osteocytes to produce M-CSF [104].

Osteocytes are embedded in the bone matrix, which raises the question of how RANKL produced by osteocytes induces osteoclast differentiation on the bone surface. The precursor of osteoclasts and osteocytes attached within the collagen gel demonstrated that RANKL originates from osteocytes and is delivered to osteoclast precursor mainly via a membrane-bound form employing the dendritic processes of osteocytes in the lacunocanalicular system. In contrast, osteocyte-producing soluble RANKL has a minor role in osteoclastogenesis [105]. Moreover, compressive forces, including orthodontic tooth movement, stimulate osteocyte-mediated osteoclastogenesis through autophagy-mediated RANKL secretion via the transcription factor E3-related signaling [106,107]. Specific removal of osteocyte-derived RANKL inhibits periodontal bone loss in standard and diabetic mice demonstrating that osteocytes play an essential role in the progression of periodontitis [108]. This research has caused a paradigm shift by defining osteocytes as a central RANKL resource.

### 3.4. Periodontitis Induces Osteocyte Apoptosis Leading to Osteoclastogenesis

Periodontal tissue cells are constantly exposed to microbial pathogens and might undergo cell death [109]. Cell death is the final cellular determination reached after complicated cellular communications and is essential to maintaining homeostasis. The cell death system is generally classified as programmed cell death (PCD) or non-PCD regarding their signal dependency. PCD is divided into two types—apoptotic and non-apoptotic. Apoptosis is cell death characterized by membrane blebbing, cell shrinkage, organelle loss, DNA condensation, and fragmentation [110]. Apoptosis has many purposes, including the elimination of non-functional cells, additional cells, and malignant cells and controlling tissue size, including bone. Osteoblast and osteoclast apoptosis play a role in bone homeostasis [111].

Stimulation of osteocytes with bacteria and inflammatory cytokines induces osteocyte apoptosis in periodontitis. Gingipain, a toxic proteolytic enzyme derived from *P. gingivalis*, degrades integrin β1 on the cell membrane and suppresses the Rho family of GTPases depolymerizes cytoskeletal protein F-actin leading to reduced cell-cell adhesion to the ECM and osteocyte apoptosis [112]. Moreover, increased proinflammatory cytokines change the osteocyte mechano-sensitivity, induce osteocyte apoptosis, and upregulate the expression of osteocyte-derived inflammatory cytokines and signaling molecules under inflammatory conditions [113]. 

Osteocyte apoptosis recruits osteoclasts toward apoptosis sites leading to osteoclastogenesis and bone remodeling [114]. The consequence of osteocyte apoptosis contributes to the secretion of several inflammatory cytokines [93,113,115], such as IL-6 [116], resulting in increased RANKL expression in osteocytes [102,103]. Apoptotic osteocytes also release adenosine triphosphate (ATP) via the activated pannexin 1 channel, which acts as the bone lineage cells through ATP receptor gated (P2) channels. This interaction upregulates RANKL expression from nearby surface bone lineage cells to aggregate macrophages to differentiate themselves into osteoclasts [117,118]. Dying osteocytes or apoptotic bodies of osteocytes can directly control osteoclastogenesis and bone remodeling by secreting RANKL [119]. Moreover, apoptotic bodies of osteocytes interact with specific markers on osteoclast precursor cells to promote TNF-α gene expression leading to osteoclastogenesis [120]. The prolonged apoptotic bodies of osteocytes can proceed to secondary apoptosis allowing the cell to secrete various inflammatory cytokines and subsequently activate immune cells to upregulate RANKL expression leading to osteoclastogenesis [121]. The function of the different types of cell death in periodontitis (apoptosis, autophagy, necroptosis, and non-PCD) deserve more exploration. Uncovering the role of apoptosis of osteocytes in the inflammatory condition in periodontitis will lead to a thorough comprehension of periodontitis occurrence and progression, which will support the establishment of future therapeutic prevention, assessment, and management of periodontitis.

### 3.5. Tissue-Specific Immunity Concept

Recently, the novel concept of “tissue-specific immunity” was introduced to emphasize the roles of local proteins in immunological processes. This theory believes that peripheral tissues and organs are not simply passive immune response targets; instead, they inherently regulate and affect immune function [121,122]. Immune cells require modification to the prerequisites and conditions of the location where these cells are produced or recruited to preserve or rejuvenate their balanced function in tissues. This adaptability regarding the intimate interaction within the tissues is called “functional immune plasticity” [122,123]. Peripheral tissues inherently obtain regulatory activity and produce homeostasis molecules to manipulate the functional plasticity of immune cells to control tissue homeostasis. The discovery of tissue-derived homeostatic molecules as the functional local protein controlling immunological plasticity is critical for comprehension of the internal potential of many organs on immune homeostasis [121]. Studying local regulatory mechanisms and uncovering novel proteins supporting functional immunological plasticity is important to better understand immune-driven inflammatory disorders, such as periodontitis. Consequently, local proteins promoting periodontal regeneration were identified to enable periodontitis treatment without synthetic compounds and antibiotics.

## 4. Periodontitis: Treatment

### 4.1. Complement

*P. gingivalis* fails to cause periodontal bone loss in germ-free mice; however, it changes the periodontal microbiota and induces destructive inflammation in conventional SPF mice [6,124]. In contrast, C5a receptor-knockout mice show no bone loss from periodontitis. Maekawa et al. suggested that *P. gingivalis* manipulates the host-protective pathway, which activates small inflammatory responses leading to chronic inflammation. Interestingly, C5- and C3-deficient mice were protected against three types of disease models, namely ligature-induced periodontitis, periodontitis caused by *P. gingivalis* inoculation, and naturally occurring periodontitis [72,76]. Therefore, uncontrolled complement activation results in severe periodontitis and bone loss. A third-generation compstatin (Cp40) analog (inhibitor peptide of C3a receptor) prevents periodontitis in mice and non-human primates. Cp40 was developed for human clinical use as AMY-101 [124] with a phase IIa clinical study showing that C3 inhibition resolves gingival inflammation and bone loss [125].

### 4.2. Periostin

Osteoblast-specific factor 2 is a matricellular protein, initially discovered in mice osteoblasts. This protein was renamed “periostin” in 1999 by Horiuchi et al. because it accumulates in the periodontal ligament and periosteum. Matricellular proteins are ECM proteins and primarily function as modulators or adapter molecules for cell-matrix interactions [126]. Therefore, periostin is thought to be involved in ECM arrangement and interacts with other adhesion molecules during periodontal healing. Periostin sources are collagen-rich connective tissues bearing mechanical force including periodontal ligament and periosteum. This protein is also associated with cell remodeling processes, such as adhesion, fibrogenesis, cell survival, and cellular differentiation [127]. Periostin derived from PDL cells induces the recruitment of human mesenchymal stem cells in vitro using the αvβ3 integrin/FAK/PI3K/Akt pathway [128]. Furthermore, periostin stimulates the migration and proliferation of human PDL cells via the PI3K/Akt/mTOR pathway during inflammatory conditions [129]. Moreover, it upregulates the expression of osteoblast-related factors RUX2 (runt-related transcription factor 2), alkaline phosphatase, and osteocalcin genes in mice osteoblast precursors in vitro [130]. Consequently, periostin can be one of the critical regulators in periodontal regeneration. Its versatility to control the function and quantity of PDL cells and osteoblasts plays an essential function in tissue remodeling related to the periodontium, which culminates in periodontal regeneration.

### 4.3. Exendin-4 (EX-4)

Exendin-4 or EX-4 has the same function as glucagon-like peptide-1 (GLP-1) in mammals. It is a GLP-1 receptor agonist mainly utilized for treating diabetic patients to control glucose levels [131]. EX-4 is speculated to be the active compound promoting periodontal regeneration. Several studies have uncovered the effects of this protein on bone MSCs (BMSCs) and periodontal ligament stem cells (PDLSCs) resulting in healing and regeneration. BMSCs from rats challenged with EX-4 upregulate osteoblast-related factors, RUNX2 and alkaline phosphatase (ALP), which promotes osteogenic differentiation into osteoblasts [132]. EX-4 alleviates the inhibitory effects of high glucose on cellular proliferation and osteogenic differentiation in PDLSCs [133]. Moreover, it stimulates osteogenic differentiation via the inhibition of NF-κB signaling and regulation of Wnt signaling [134] and enhances bone mineral density in the femoral bone by decreasing the expression of SOST/sclerostin in type 2 diabetic OLETF rats by acting on osteocyte GLP-1 receptors in vivo [135]. These positive roles of EX-4 on BMSCs and PDLSCs can pave the way for future studies using this compound to promote periodontal regeneration in periodontitis patients.

### 4.4. Developmental Endothelial Locus-1 (DEL-1)

DEL-1 is the local protein mainly produced from specific resident cells in many tissues including endothelial cells, osteolineage cells, MSCs, and specific macrophage phenotypes [121]. DEL-1 interacts with β2 integrins and αv integrins, and phospholipids, which enables it to serve as a local and tissue-derived immunomodulator. DEL-1 locally inhibits the recruitment of neutrophils to the area of inflammation by blocking the lymphocyte function-associated antigen-1 found on neutrophil surfaces to the intercellular adhesion molecule-1 exposed on endothelial cells [136,137]. DEL-1 is also related to the resolution period and bone regeneration process and promotes induction and suppressive activity of RUNX1-dependent FOXP3 in human T cells [138]. Furthermore, DEL-1 promotes osteoblastogenesis and new bone formation through its RGD motif, which involves the αvβ3–FAK–ERK1/2–RUNX2 signaling pathway [139]. DEL-1 also works as a central regulator of immune homeostasis and is a significant protein in the bone marrow niche that induces an emergency myelopoiesis for host immunity [140]. These abilities allow DEL-1 to control the immune responses, which reveal the remarkable effects in suppressing osteoclasts, alleviating inflammation, and promoting osteoblastogenesis in the periodontitis-induced mouse model [141,142,143] and non-human primates in animal studies [144]. DEL-1 is an anticipated periodontitis therapeutic agent against periodontitis, which removes the dependence on antimicrobial agents [145].

### 4.5. Synthetic and Natural Compounds That Alleviate Periodontitis

Rice protein peptides inhibited the transcriptional activity of inflammatory and osteoclast-related molecules in a mouse periodontitis model and in vitro [146]. Proanthocyanin is a potent grape seed antioxidant, which was reported to decrease inflammation and periodontitis-induced alveolar bone loss by reducing HIF-1α and MMP-8 levels and increasing the activity of osteoblast cells in periodontitis-induced diabetic rats [147]. *Spirulina maxima*, a cyanobacterium, which has anti-inflammatory properties in a gastric ulcer model [148], also reduces proinflammatory cytokines, suppresses osteoclastogenesis, and upregulates the BMP-2/Smad pathway and osteogenesis-related factors in periodontitis-induced mice [149]. Synthetic salubrinal ameliorates periodontitis by reducing alveolar bone loss and inflammatory cytokines and induces bone formation in the periodontitis-induced mouse model [150]. *Zanthoxylum piperitum* DC (ZP) was initially used as a food condiment and exhibits therapeutic effects including the prevention of toothache [151]. Topical treatment with ZP dried fruit extract inhibited osteoclast function and alveolar bone loss due to periodontitis in an animal study. Moreover, ZP reduced RANKL and upregulated RUNX2 and osterix gene expression in SaOS-2 osteoblasts [152]. Each of these agents ameliorates periodontitis inflammation and could be a novel therapeutic option for treating patients suffering from this disease in the future.

## 5. Closing Remarks and Perspectives

Osteocytes, the most prevalent cell type in the bone, outlast all other bone cells. They interact via a network of dendrites that resembles an organ, allowing osteocytes to control the inflammatory process locally and systemically. Osteocytes behave as endocrine cells by secreting substances that impact the osteocytes themselves, the immediate region of the osteocyte, or the entire body [83]. Osteocytes respond to various effector cytokines by producing additional cytokines, which help reduce or stop inflammation and bone resorption. Previously assumed to be inactive, osteocytes are increasingly gaining attention and should be evaluated as target cells for therapy of bone diseases. Osteocytes perform duties once considered to be reserved for other specialized cell types. Future research might reveal more about the osteocyte-bone dynamics and confirm osteocytes as target cells for medication to treat bone resorption and other systemic inflammatory illnesses.

Keystones in periodontitis onset and disease progression are biofilm dysbiosis coupled with sustained inflammation in the periodontium [7]. Presumptive periodontal pathogens are essential for provoking inflammatory responses and perpetuating disease by disturbing immune responses [6,8]. While these concepts provide a standpoint for considering the roles of microbiota in periodontal disease, significant controversies remain; it could plausibly be concluded that bacteria cause gingivitis because their clearance results in the healing of this inflammatory condition. However, the factors that may influence the dysbiosis of microbial biofilm resulting in the progression from gingivitis to periodontitis are less well established. Even though dysbiosis relates to periodontitis [65,153], it is still unclear whether dysbiosis originates, co-develops, or develops after the disease occurs. However, the relationship between inflammation and dysbiosis is significant [7,48]. Correspondingly, molecular studies of virulence factors from the microbiota driving the destructive inflammatory response are currently inconclusive despite considerable data, mainly derived from in vitro studies, implicating several virulence factors, albeit merely in the limited number of numerous periodontitis-associated pathogens that have already been thoroughly examined as of date [48]. 

The advancement of omics technologies (the universal detection of a specific biological specimen targeting genes [genomics], mRNA [transcriptomics], proteins [proteomics], etc.), and enhanced database collection together with bioinformatics has been aiding the discovery of other active microbial crosstalks and related products, for example, pathogenic genes, novel signaling proteins, and their metabolites stipulated to be involved in disease progression [154]. Integrating omics data with established data about the pathogenicity of microorganisms and supplementary in vivo polymicrobial models could be an effective method for identifying relevant disease-associated microorganisms, virulence factors, and mutual interchanges that affect the composition of the bacterial biofilm and host immune responses [155]. As a result, a more comprehensive range of therapeutic targets can be identified, allowing for the development of better, precise, and effective therapeutic approaches. Treatment approaches that suppress the inflammatory reactions leading to tissue damage or accelerate the resolution phase of inflammation could offer alternative therapeutic approaches apart from antimicrobial drugs currently prevalent in dental practice.

Overall, studies on dysbiosis of oral microbiota causing inflammation and novel discoveries of agents and proteins for therapeutic approaches have drawn our interest to the era of specific-targeted treatment approaches for periodontitis related to the control of immune responses via the use of specific agents or proteins. Novel therapeutic modalities related to periodontitis-related pathways or signaling molecules have been studied and aimed to be used as therapeutic options aside from the traditional antibiotic treatments. The previously mentioned proteins and synthetic agents have been studied for their effects on the periodontium and are stipulated to be optional drugs for treating periodontitis. Incorporating novel concepts of immunomodulation into the existing concepts of pathogenesis will highlight the importance of immune cells, which could further impact periodontitis and other inflammation-driven diseases.

## Data Availability

Not applicable.
